# The Varieties of Neurasthenia

**Published:** 1909-07-31

**Authors:** 


### THE VARIETIES OF NEURASTHENIA.

The term neurasthenia was originally introduced by Beard to denote that which was at first regarded as a distinct disease entity, but is now known to be merely a convenient means of designating a symptom group, which may be observed under more than one series of conditions. It is a term, moreover, that is arbitrarily restricted in its meaning, for by no means every state in which there is deficiency of nerve power or of nervous force can be styled neurasthenic. There is an immense reduction in the nervous power of individuals who are obviously suffering from typhoid fever, for example, or from cancer of the stomach; but it would detract greatly from the clinical value of the term neurasthenia if it were applied to such cases as these. Similarly, even in cases of chronic ill-health, in which there are no very definite symptoms or signs, it is wise to avoid any hasty diagnosis of neurasthenia; for the latter term should be reserved for condition of deficient nervous energy for which no definite post-mortem changes would be found. In a young person gradual loss of energy and strength is much more likely to point to commencing phthisis than to neurasthenia alone, and in an individual of maturer years such lesions as arteriosclerosis, granular kidney, or an early stage of cancer of the stomach must be excluded before a diagnosis of neurasthenia is made.

Even so, however, there remain undoubted cases to which the term in its restricted sense is applicable. The syndrome is familiar enough, the peculiar feelings in the head, or elsewhere, the headaches, the dyspepsia, the insomnia, the ready fatigue, both of body and of mind, the impairment of memory, the difficulty in maintaining concentrated attention, the mental indecision, the occasional panics. Without entering into a discussion of the different types that have been described, according to the part of the nervous system most affected—the cerebral, the spinal, the cerebrospinal, the sympathetic or visceral types—there can be no doubt that the same symptom groups can be produced by different causes; and if the treatment is to be successful it is necessary to adopt very different measures in different cases. Classified according to causation, one may distinguish at least the following types of neurasthenia:—

(1). A neurasthenia due to the excessive expenditure of nerve force over considerable periods of time. This is seen particularly in two classes of patients. First, those who come of "nervous" families, and who have, therefore, inherited nervous systems which can sustain comparatively slight nervous expenditure; and, secondly, those who, having inherited a particularly strong nervous system, have used it to the very utmost in business, or otherwise, until the expenditure of nervous force has exceeded the nervous income, and there is a nervous bankruptcy—neurasthenia. The latter class of case occurs mainly amongst those whose interests have been centred upon a comparatively small number of objects—money-making as an aim for example, especially in the person who has no hobby outside his work. It is good for the nervous system to have variety. All work and no play makes Jack a dull boy; it produces neurasthenia.

(2). A neurasthenia due to injury. The railway spine is a familiar example of a condition which lies on the borderland between true neurasthenia in its restricted sense, and an organic lesion resulting from multiple small spinal hæmorrhages. It will always be a matter of the greatest difficulty to assess, in a given case, the precise degree to which some of the symptoms that result from injuries to the head or back are due to gross changes in the central nervous system, on the one hand, or to neurasthenia proper on the other.

(3). A neurasthenia that results from the chronic mis-use of such things as tobacco, alcohol, or tea. It is not always easy to decide whether it is the substance mis-used, or the circumstances under which it is misused, that is most responsible for the symptoms, but that it is the substance rather than the circumstances in many instances is exemplified by the greater ill-effects that follow the use of some kinds of cigarettes than others.

(4). A neurasthenia that results from too monotonous and sedentary an indoor mode of living, seen especially in women in towns. The nerve exhaustion results in these cases less from too great an expenditure than from too little cultivation of the nervous tree. Nervous exhaustion also plays a part, arising from worries and anxieties in connection with the illnesses of children, money matters, and so on.

(5). A neurasthenia which is the first stage of melancholia, or of some other mental disease, such as general paralysis of the insane.

(6). A neurasthenia due to auto-intoxication from the intestines. It is the latter variety of neurasthenia that receives not a little benefit from lactic acid bacterial treatment, though Mr. Arbuthnot Lane, it seems, would advocate colectomy as the proper cure.

